# Unexpected Diagnosis of Both Adenocarcinoma of the Colon and Metastatic Lobular Carcinoma of the Breast in the Gastrointestinal Tract

**DOI:** 10.1155/2013/153180

**Published:** 2013-07-24

**Authors:** Tegan Miller, Carol Ross, Haitham Al-Rawi, Barry Taylor, Mohammad Al-Jafari

**Affiliations:** ^1^Histopathology Department, Warrington and Halton Hospitals NHS Foundation Trust, Lovely Lane, Warrington WA5 1QG, UK; ^2^Surgical Department, Warrington and Halton Hospitals NHS Foundation Trust, Lovely Lane, Warrington WA5 1QG, UK

## Abstract

Breast cancer rarely metastasises to the gastrointestinal tract. Lobular carcinoma more commonly metastasises to the uterus and appendages, peritoneum, and gastrointestinal tract than other types of breast cancer, while ductal carcinoma has a propensity to metastasise to the lungs, liver, and brain. We describe the case of a patient with no known history of breast cancer, whose primary presentation of lobular breast cancer was with malignant small intestinal and colonic strictures, with coexisting previously undiagnosed adenocarcinoma of the colon.

## 1. Introduction

Breast cancer is the commonest malignancy in women and comprises 27% of all cancers [1, 2]. The introduction of the national breast screening programme has improved earlier detection, and only 7% of breast malignancies have metastasized at the time of presentation [1]. Lobular and ductal carcinomas of the breast both originate from the terminal ductal-lobular unit but have very different metastatic patterns [3]. In clinical practice, breast cancer rarely metastasises to the gastrointestinal tract, and this is usually associated with disseminated disease [1, 4, 5]. Lobular carcinoma is known to spread to the gastrointestinal tract disproportionately more commonly when compared with other breast cancers [6]. We describe the case of a patient with no known history of breast cancer, whose initial presentation of lobular breast carcinoma was with malignant colonic strictures, with coexisting previously undiagnosed adenocarcinoma of the colon.

## 2. Materials and Methods

A 78-year-old lady presented to the outpatient department with weight loss, left iliac fossa pain, and constipation. She had a past medical history of hypertension, gastroesophageal reflux disease, gallstones, a transient ischaemic attack in 1998, and a nodular basal cell carcinoma excised in 2006. Abdominal examination was unremarkable. Computed tomography colonography was performed, which showed a 1.5 cm stricture in the distal sigmoid colon and multiple small sclerotic foci throughout the skeleton. A bone scan showed degenerative changes and no evidence of metastases. Following two unsuccessful endoscopies due to inability to pass the scope past the stricture, a laparotomy and subtotal colectomy were performed. In theatre, the patient was noted to have two strictures, one in the sigmoid and one in the right colon combined with features of severe retroperitoneal fibrosis. Following surgery, she was treated at the Intensive Care Unit for postoperative hypotension. She was later transferred to the ward and discharged home following a good recovery.

The subtotal colectomy specimen measured 660 mm in length, including 120 mm of terminal ileum with attached appendix. Three strictures were noted: one in the ascending colon, another in the middle of the transverse colon, and the third in the sigmoid colon. 

**Figure 1:**
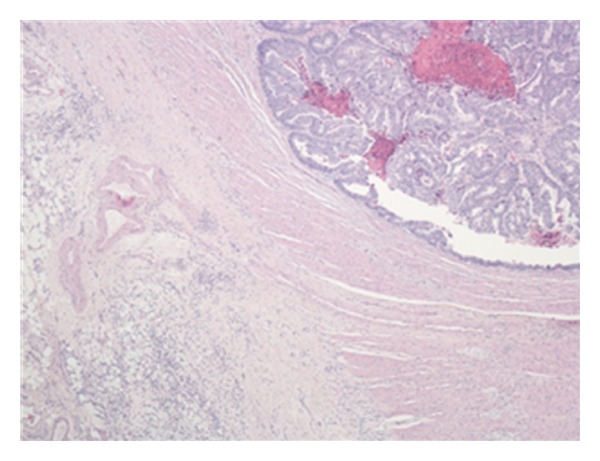
Low power light microscopy image demonstrating well-differentiated adenocarcinoma (upper right of image) and small noncohesive cells with the appearance of metastatic infiltration (lower left of image).

Microscopic examination of the sigmoid stricture revealed moderately differentiated adenocarcinoma (Figure 1), extending beyond the muscularis propria. A few foci of extramural vascular invasion were demonstrated, and one out of nine lymph nodes contained metastatic adenocarcinoma. The apical node was negative. The TNM staging was pT3 pN1 pMx, and Dukes stage was C1.

**Figure 2:**
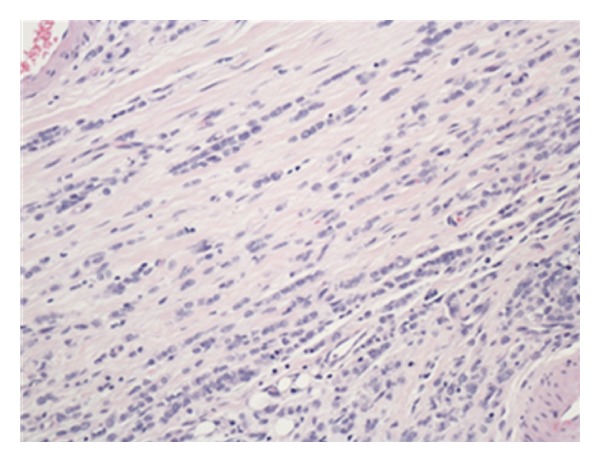
High power light microscopy image of metastatic cells showing the classical appearance of lobular carcinoma of the breast with discohesive cells forming an “Indian file.”

**Figure 3:**
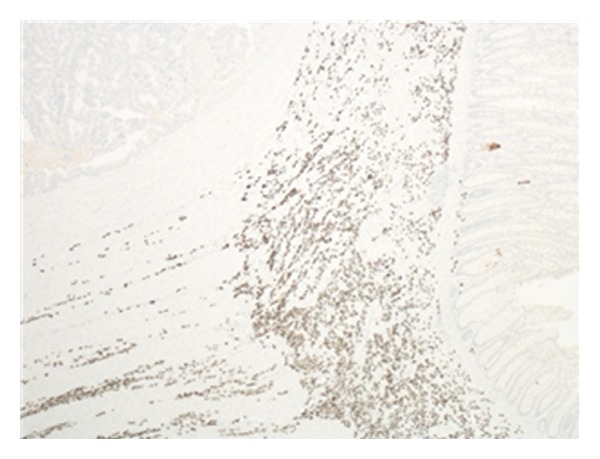
Immunohistochemical staining with CK 7 demonstrating negative adenocarcinoma uptake and positive metastatic carcinoma uptake.

The walls of the large and small intestines, including the ascending and transverse strictures noted earlier, were extensively infiltrated by metastatic tumour which was composed of diffuse discohesive small, rather uniform, cells (Figures 1 and 2). These cells showed a single file appearance known as “Indian filing.” Several immunohistochemical stains were performed which most notably showed positive staining for cytokeratin (CK) 7 (Figure 3) and estrogen receptor (ER). These cells were negative for CK20, CD45, and CEA. All of the nine lymph nodes sampled were involved by this metastatic tumour. These findings were consistent with metastatic lobular carcinoma of the breast with a simultaneous primary colonic adenocarcinoma.

As a result of this diagnosis, the patient was referred to oncology services and received hormonal therapy for the metastatic breast cancer. The patient is currently under the care of the palliative care team, four months after diagnosis. Further imaging had not located any primary breast cancer.

## 3. Discussion

The commonly described metastatic sites of lobular carcinoma of the breast vary from those of ductal carcinoma [6]. Lobular carcinoma more commonly metastasises to the uterus and appendages, gastrointestinal tract, and peritoneum [7, 8], whereas ductal carcinoma has a propensity to metastasise to the lungs, liver, and brain [8]. Although rarely detected clinically, a postmortem series has suggested that between 8 and 35% of breast cancers metastasised to the gastrointestinal tract [2, 7, 9]. The stomach and small intestine are more commonly involved in metastatic breast carcinoma than the colon or rectum, the latter two being only very rarely involved [8]. Gastrointestinal involvement of any type of breast cancer is rarely suspected clinically, and diagnosis can be more difficult as symptoms can be nonspecific or mimic those of a primary gastrointestinal tumour [7, 8]. Abdominal pain and bloating have been found to be the most commonly reported symptoms by patients who were found to have gastrointestinal metastatic deposits of breast cancer [8].

Gastrointestinal metastases of lobular breast carcinoma are very rarely a first presentation of breast cancer [8]. Our literature review has revealed only two previous small bowel [10, 11] and one previous colorectal [12] resection as the location for metastatic deposits of undiagnosed lobular carcinoma of the breast, as was found in this case. Calò et al. reported a case of bowel obstruction due to a solitary jejunal metastasis of previously undiagnosed lobular carcinoma of the breast [10]. Sato et al. presented a case of a patient with small bowel obstruction, who was found to have duodenal metastases of lobular carcinoma of the breast with no clinical manifestations of breast cancer [11]. Arrangoiz et al. described a case of a patient presenting with partial large bowel obstruction, who was found to have rectal, peritoneal, and gastric metastases from undiagnosed lobular carcinoma of the breast [12]. Three case reports describe gastric metastases [4, 8, 13], and one case report found intraperitoneal metastases [14] as the first presentation of lobular carcinoma of the breast. When considering gastrointestinal metastases of lobular carcinoma, the stomach is more commonly involved than other gastrointestinal organs [2]. The literature search did not reveal any other cases of coexisting metastatic lobular carcinoma with any other primary malignancy, as was found in this case.

The histological appearance of lobular carcinoma of the breast differs from that of ductal carcinoma, with invasive lobular cancer appearing as small cells which infiltrate with a single “Indian file” appearance [15]. Immunohistochemistry has an established role in the diagnosis of cancer [3]. Estrogen receptor (ER) is expressed in 70–75% of breast cancers, giving a high sensitivity for the diagnosis of such cases [3, 5]. Likewise, cytokeratin 7 is highly sensitive for breast cancer but is expressed in many epithelial tissues [3]. Cytokeratin 20 is not expressed in breast cancers but is found in pancreatic, gastric, and colorectal cancers, which aided diagnosis of adenocarcinoma of the colon in this case [3, 6].

Accurate diagnosis enabled prognostic information to be provided to the patient, as well as appropriate access to cancer services. Although chemotherapy was not appropriate in this case, accurate diagnosis enabled hormonal therapy to be commenced.

## 4. Conclusion

Lobular carcinoma of the breast rarely metastasises to the gastrointestinal tract and these metastases are particularly a rare first presentation of breast cancer [1, 4]. In addition, a coexisting primary cancer has not been reported with metastatic lobular breast cancer. Diagnosis of gastrointestinal metastases of breast cancer can be difficult due to the rare occurrence and the vague symptoms with which it presents [7]. Knowledge of the metastatic patterns of breast cancer by clinicians and pathologists together with the utilisation of immunohistochemical stains is essential in establishing an accurate diagnosis, assessing prognosis, and planing further therapy.
